# Effect of Silver or Copper Nanoparticles-Dispersed Silane Coatings on Biofilm Formation in Cooling Water Systems

**DOI:** 10.3390/ma9080632

**Published:** 2016-07-29

**Authors:** Akiko Ogawa, Hideyuki Kanematsu, Katsuhiko Sano, Yoshiyuki Sakai, Kunimitsu Ishida, Iwona B. Beech, Osamu Suzuki, Toshihiro Tanaka

**Affiliations:** 1 Department of Chemistry and Biochemistry, National Institute of Technology, Suzuka College, Suzuka 510-0294, Japan; 2 Department of Materials Science and Engineering, National Institute of Technology, Suzuka College, Suzuka 510-0294, Japan; kanemats@mse.suzuka-ct.ac.jp; 3 D & D Corporation Ltd., Yokkaichi 512-1211, Japan; sano@ddcorp.co.jp; 4 Department of Maritime Technology, National Institute of Technology, Toba College, Toba 517-8501, Japan; y-sakai@toba-cmt.ac.jp (Y.S.); ishida@toba-cmt.ac.jp (K.I.); suzuki@toba-cmt.ac.jp (O.S.); 5 Biocorrosion Center, University of Oklahoma, Norman, OK 73019, USA; ibeech@ou.edu; 6 Division of Materials and Manufacturing Science, Graduate School of Engineering, Osaka University, Suita 565-0871, Japan; tanaka@mat.eng.osaka-u.ac.jp

**Keywords:** cooling pipe, biofilm, silver nanoparticle, copper nanoparticle, silane coating, bacterial taxonomy, seawater, geometry, Raman spectroscopy

## Abstract

Biofouling often occurs in cooling water systems, resulting in the reduction of heat exchange efficiency and corrosion of the cooling pipes, which raises the running costs. Therefore, controlling biofouling is very important. To regulate biofouling, we focus on the formation of biofilm, which is the early step of biofouling. In this study, we investigated whether silver or copper nanoparticles-dispersed silane coatings inhibited biofilm formation in cooling systems. We developed a closed laboratory biofilm reactor as a model of a cooling pipe and used seawater as a model for cooling water. Silver or copper nanoparticles-dispersed silane coating (Ag coating and Cu coating) coupons were soaked in seawater, and the seawater was circulated in the laboratory biofilm reactor for several days to create biofilms. Three-dimensional images of the surface showed that sea-island-like structures were formed on silane coatings and low concentration Cu coating, whereas nothing was formed on high concentration Cu coatings and low concentration Ag coating. The sea-island-like structures were analyzed by Raman spectroscopy to estimate the components of the biofilm. We found that both the Cu coating and Ag coating were effective methods to inhibit biofilm formation in cooling pipes.

## 1. Introduction

Cooling systems significantly influence the energy conversion efficiency of chemical plants and thermal power plants. Cooling systems are classified as either wet or dry. Wet type cooling systems use water or other liquid solutions as the heat transfer medium, whereas dry ones use air. Many plants adopt water cooling systems because water has a higher heat efficiency than air. In water cooling systems, natural water such as seawater and river water is used as the heat transfer medium because this system needs a lot of water. For example, 11 million liters of water per day are used in a nuclear power plant [1]. Such natural water is abundant in microbes and minerals, which causes biofouling in cooling systems, resulting in corrosion on the surface of the cooling pipes and reduction in the thermal transfer efficiency [2]. Venugopalan et al. reported that a cooling water system will cost more than US$30,000,000 to repair or replace it in a large nuclear power plant. In addition, 20% of the total corrosion damage is caused or influenced by microbes in the heat exchangers [3]. This type of corrosion is called microbially influenced corrosion (MIC).

The initial stage of MIC is the formation of a conditioning film. A conditioning film consists of not only organic chemical compounds but also non-organic ones. Conditioning films attract microbes of natural water. These microbes attach on the conditioning film and then grow, multiply and produce extracellular polymeric substances (EPSs), resulting in a biofilm. Once a biofilm is formed on the surface, the inside of the biofilm will differ in ion and oxygen concentrations from the outer biofilm, which will trigger corrosion on the surface of the cooling pipes. This is why controlling (regulating) biofilm formation is very important. In this study, we modified the surface of cooling pipes by coating to delay or reduce the biofilm formation. The coating method has some advantages: treatment is easer and the cost is lower than synthesizing new pipe materials.

Biofilms are generally considered as consisting of bacteria and EPSs such as polysaccharides, proteins and extracellular DNA [4,5,6]. At the very early stage of biofilm formation, the attached bacteria on the surface of materials multiply and produce EPSs. However, biofilms grow incorporating much matter from environments in the following stage of biofilm formation. Seen from the viewpoint of effects on materials, the latter one is the most important. Generally, researchers consider that the complex of the very early stage is a biofilm, and they have mainly analyzed and quantified the bacteria related to biofilm by scanning electron microscopic images, crystal violet staining, and so on [7]. Meanwhile, we consider that both complexes of the very early stage and that of the following stage are biofilms in broad sense. In order to analyze the biofilm, EPSs and organic compounds from environments are key factors. Raman spectroscopy is a useful analytical method for organic compounds such as hydrocarbons, nucleic acids, proteins etc. [8,9,10,11]. In addition, some researchers have recently analyzed biofilm by Raman spectroscopy [12,13,14,15], and we succeeded in observing biofilms formed on composite coated iron by Raman spectroscopy in our previous study [16]. Therefore, we applied Raman spectroscopy for identification of biofilm here.

In our previous study, organic metal conjugated silane-based polymer coatings affected biofilm formation and silver acetate conjugated silane-based polymer coatings significantly reduced biofilm formation [17,18]. In this study, we investigated the use of silver nanoparticles-dispersed silane-based polymer coatings to prevent biofouling. In addition, we also investigated the use of copper nanoparticles because some researchers have reported that copper has antimicrobial activity against some microbes such as *Salmonella*, *Campylobacter* and *Mycobacterium* [19,20]. The silane based polymer has nano-order holes [21] where silver or copper nanoparticles will be captured. Therefore, we will be able to control the elution speed of metals from the polymer by changing the size of nanoparticles, which results in the effect of the metals on anti-biofilm formation lasting. In addition, we expected that metal nanoparticles dispersed silane polymers will have an easier time producing metallic ions for controlling the biofilm in comparison with the organo-metal conjugated silane coating, and that higher environmental safety would be achieved to avoid potentially dangerous organo-metals.

We used a closed laboratory biofilm reactor (LBR) and Ise Bay (IB) seawater from Japan as a model for the cooling water pipe and cooling water, respectively. After we performed the biofilm formation tests in the LBR system, we removed each coupon and analyzed the surface by three-dimensional digital microscopy to create a roughness image, and used Raman spectroscopy to confirm whether the sediment formed on the surface is a biofilm. We also analyzed the bacterial content of IB seawater based on the next generation sequence technique using the *16S rRNA* gene [22] to give us useful information about which bacteria in IB seawater could contribute to biofouling in the cooling pipe system.

## 2. Materials and Methods

### 2.1. Specimens

Stainless steel SUS304 sheet (1 mm thickness) was purchased from Nilaco Co., (Tokyo, Japan). Soda lime float plate glass (1 mm thickness) was used as a basal plate (Central Glass Co., Tokyo, Japan) for silane-based polymer coatings. These plates were cut into 10 × 20 mm^2^ pieces by a handheld diamond cutter and then a hole was made at both ends of the short side. They were washed with acetone, and then dried for 24 h in a desiccator before the coating process. The silane-based polymer coating was prepared by mixing 20-g zirconia beads (1 mm diameter) with two oligomers; A: alkoxysilane oligomer containing methyl and phenyl functional groups, named Permeate (MW 360, D & D Co., Yokkaichi, Japan) and B: n-2-(aminomethyl)-3-aminopropyltrimethoxysilane, named KBM-603 (MW 222, Shin-Etsu chemical Co., Tokyo, Japan). The weight ratio of oligomer A to B was 80% to 20%. The mixing was performed in a 250-mL polypropylene bottle injected with nitrogen air using an agitator (Toyobo, Osaka, Japan) for 30 min. Silver nanoparticles or copper nanoparticles (Sigma-Aldrich, St. Louis, MO, USA) were dispersed at the same time as mixing of the oligomers. The silver and copper nanoparticles were 100 nm and 40–60 nm in diameter, respectively. After 2 h, the adjusted coating solution was filtered through a nylon mesh #110 (NBC Meshtec Inc., Hino, Japan) to remove any residue and impurities. The filtered coating solution was sprayed on the surface of each glass coupon or each stainless coupon then incubated at 20 °C for 7 days to solidify the coating.

### 2.2. Sampling Seawater

**Figure 1:**
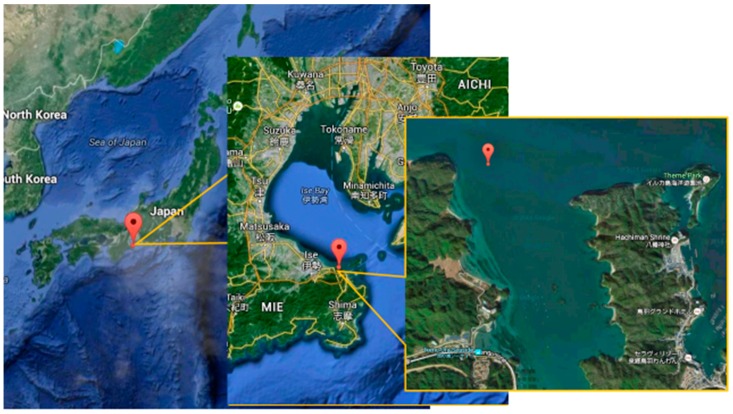
Sampling point of Ise Bay seawater. The **red** balloon shows the sampling point. This map is from Google Maps (Toba, Mie, Japan).

We sampled seawater from one part of Ise Bay in Japan (Figure 1) to avoid human sewage and agricultural drain. The exact sampling point was the entry of the Ikenoura inlet (34°30.4533′N, 136°48.8626′E). The depth at this location was about 50 m and the distance was 302 m from the land. We dropped the autoclaved media bottles (1 L) from the boat using a 2.2-m length of rope tied to the mouth and collected seawater at a depth of 2 m from the ocean surface. After raising the bottles out of the water, they were closed immediately and covered with a double layer of aluminum foil to prevent influence from light. The bottles were kept in a refrigerator (10 °C) until the biofilm formation experiments.

### 2.3. Biofilm Formation

**Figure 2:**
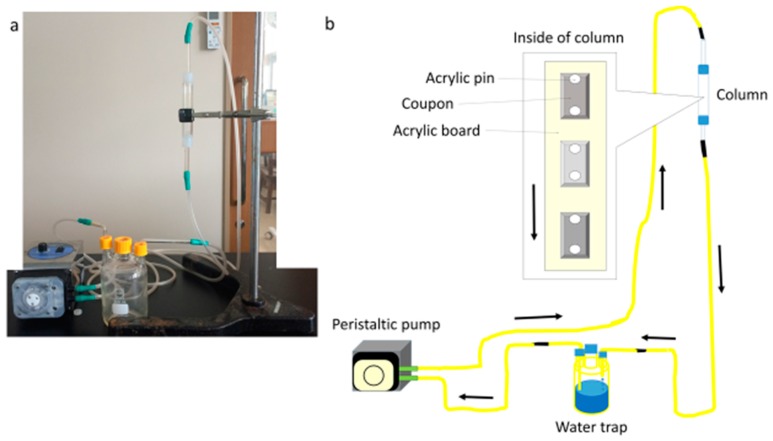
Schematic representation of a closed laboratory biofilm reactor. (**a**) photo; (**b**) illustration. The **black** arrows indicate the direction of the water flow.

Coated SUS304 or coated glass coupons (D & D Co., Yokkaichi, Japan) were sterilized by dipping them in 70% ethanol solution for 10 min. Two to five coupons were secured onto an acrylic board using acrylic pins. The acrylic board was placed in an acrylic column. This column was joined to silicon tubes connected to a water trap. The acrylic column and silicon tubes were filled with IB seawater or filtered seawater. The water trap bottle contained 0.3 L of IB seawater or filtered seawater (total water volume was about 0.4 L). The seawater was circulated through the silicon tubes at 0.3 L/min for 3 or 7 days using a peristaltic pump (Figure 2).

### 2.4. DNA Extraction from Seawater

**Figure 3:**
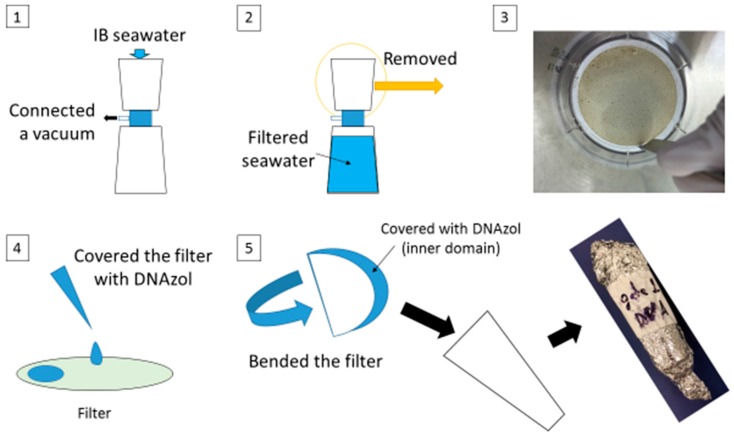
Outline of IB seawater filtration and storage.

One liter of IB seawater was loaded into a sterile disposable filter unit with a 0.1 μm pore size polyethylene sulfate membrane (Thermo Fisher Scientific, Waltham, MA USA) and filtrated by vacuum very gently. The membrane was cut off from this unit using a sterile stainless spoon. The IB seawater attached side of the filter was covered with 1 mL of DNAzol (Molecular Research Center, Cincinnati, OH, USA) and was then bent in half as the inner side was covered with DNAzol. It was transferred to a sterile centrifuge tube covered with aluminum foil and preserved at room temperature (25–30 °C) for 7 days (Figure 3). On the eighth day, DNA extraction was performed as follows: 1 mL of lysis solution, consisting of 953 μL of nuclease-free water, 37 μL of proteinase K (Thermo Fisher Scientific) and 10 μL of 10% sodium dodesylsulfate solution, was added to the tube containing the membrane and vortexed briefly. The tube was incubated for 1 h at 50 °C, and then 250 μL of RNA lysis buffer (RLA) solution, 250 μL of RNA dilution buffer (RDB) solution and 1 tube of Lysing Matrix beads E (MP Biomedicals, Santa Ana, CA, USA) were added. RLA and RDB were obtained from Maxwell^®^ 16 tissue LEV total RNA purification kit (Promega, Madison, WI, USA). Each sample was agitated by vortex (Thermo Fisher Scientific) at the highest speed for 2 min at room temperature then centrifuged at 7400× g for 8 min at room temperature. The supernatant was transferred into two cartridges of Maxwell^®^ 16 tissue LEV total RNA purification kit to purify the DNA using Maxwell^®^ 16 Research equipment (Promega). The total volume of purified DNA sample was 100 μL. The purified DNA samples were quantified using a Qubit 2.0 fluorometer (Thermo Fisher Scientific) and Qubit dsDNA HS assay kit (Thermo Fisher Scientific).

### 2.5. 16S rRNA Gene-Based Bacterial Community Analysis

Bacterial and archaebacterial *16S rRNA* genes were amplified partially using universal 16S primers: 519F (5′-CAGCMGCCGCGGTAA-3′) and 785R (5′-TACNGGGTATCTAATCC-3′). The 5′ end of 519F was tagged with M13 (5′-GTAAAACGACGGCCAG-3′, M13-519F) [23] to allow the addition of illumina^®^ primers and barcode. The polymerase chain reaction (PCR) solution (total amount of 25 μL) consisted of high-fidelity PCR master mix (NEB Labs, Ipswich, MA, USA), 0.2 μM of primers (M13-519F and 785R), 5–10 ng of extracted DNA sample and reverse transcription-polymerase chain reaction (RT-PCR) grade water (Thermo Fisher Scientifics). PCR conditions were as follows: the initial step was at 98 °C for 30 s, repeated at 98 °C for 10 s, then at 52 °C for 20 s and at 70 °C for 10 s (25 cycles) and the final step was at 70 °C for 5 min using thermal cycler (Techne, Staffordshire, UK). The PCR products were checked by gel electrophoresis to confirm that just the expected PCR amplicons were produced. The PCR amplicons were cut out from the gel and transferred to 50 μL of nuclease-free water, and then used for tagging the PCR reaction. Each PCR amplicon was tagged for MiSeq^®^ illumina^®^ sequencing by a unique 12 base pair barcode connected to 19–20 base pair linker sequence and M13 (barcode-M13). Tagging of the PCR reaction solution (total amount of 50 μL) consisted of first PCR amplicon (5 μL), 0.2 μM of barcode-M13 primer, 0.2 μM of 785R primer, PCR master mix and nuclease-free water. The tagging PCR was performed under the same conditions as the first PCR reaction except for the cycle number. After the reaction, all of the tagged PCR products were cleaned up using a magnetic beads reagent following the manufacturer’s procedure (Beckman Coulter, Brea, CA, USA). The concentrations of the cleaned samples were measured, and the samples were pooled in one tube as each DNA amount used to create the *16S rRNA* library was equal, then concentrated by Amicon^®^ Ultra 30K membrane filter units (Merck Millipore, Billerica, MA, USA). We sent the library (110 μL) to Oklahoma Medical Research Foundation (Oklahoma City, OK, USA). The *16S rRNA* gene library was pre-processed and analyzed using a combination of USEARCH version 5.2.236 and 6.1.544 [24,25], the bioinformatics software package QIIME™ version 1.9.0 [26,27] and ea-utils [28], which are fastq processing utilities. First, the forward sequence raw data file and the reverse sequence file were joined to stitch reads together using the *fastq-join* script under the condition that the maximum difference was 3% and the minimum overlap size was 50 bases [29]. Second, the barcodes and reads were extracted using the *extract_barcodes.py* script. The extracted reads were demultiplexed using the *split_libraries_fastq.py* script. The sequences were clustered based on 99% identity and an operational taxonomic unit (OTU) table was created using the *pick_de_novo_otus.py* script. The ribosomal database project (RDP) classifier [30] against the Silva 111 database [31] was used for taxonomy assignment of the bacteria. Third, chimeric sequences were identified in the demultiplexed sequences data file such a seqs_rep_set.fasta by the *identify_chimeric_seqs.py* script and usearch61 algorithm against the reference sequences of Silva 111 database (90_Silva_111_rep_set_fasta), and then removed from the original OTU table by the *filter_otus_from_otu_table.py* script. Finally, the bacterial diversity analysis was performed using the *core_diversity_analyses.py* script and an OTU table of non-chimeric sequences. The raw data files have been deposited in the National Center for Biotechnology Information (NCBI) Sequence Read Archive (SRA) and the accession number is SRP078779.

### 2.6. Three-Dimensional Image of the Surface of Coupons

After a culture, each coupon was removed from the LBR and observed by high-speed microscopy (VW-9000, Keyence Co., Osaka, Japan) to obtain a three-dimensional image of the surface of the coupons with a thousand-fold magnification. We selected randomly ten points (center, near to edges, between center and edges) of the surface area of each sample. The specimen was placed on the stage of microscope and the stage was shifted around the focal point slightly. At every step of the shift, the surface image was captured and all of them were integrated into a three-dimensional image, which the PC attached with the microscope virtually made. Colors were assigned to each place, depending on the height of places. For example, the red color was assigned to the highest place, while the blue color to the lowest one. In addition, the intermediate colors between red and blue were given to the places according to their heights. As a result, we could get the color pattern on behalf of surface profiles. The color patterns could be classified into the two types. When only the continuous gradient could be seen, we judged that the pattern would reflect only the inevitable microscopic tilt of specimens. On the other hand, we could get the inhomogeneous pattern where the place of red color was scattered on that of blue one. We judged that the pattern belonged to biofilm [32]. The microscope scanned the sections 100 times between the top and bottom then reconstructed them to create three-dimensional images in multiple colors. We checked that all ten microscopic images from the same sample showed similar roughness and shape. Each three-dimensional image in figures shows the best images of these samples.

### 2.7. Raman Spectroscopy Analysis

After microscopic observation, each coupon was freeze-dried using the following process: (1) the coupon was dehydrated by soaking in a 30%, 50%, 60%, 70%, 80%, 90%, 95%, 98%, and 99.5% ethanol solution in sequence for 15 min at room temperature; (2) the dehydrated coupon was transferred to a mixture of ethanol and t-butanol and incubated for 15 min at room temperature. The ratio of ethanol to t-butanol was 7 to 3, 5 to 5, and 3 to 7 in sequence; (3) the coupons were soaked in t-butanol and stored in a refrigerator (at 10 °C) overnight; and (4) the coupons were transferred to a desiccator and placed under vacuumed until the frozen t-butanol disappeared completely. The freeze-dried coupons were analyzed by a laser Raman spectroscopy (NRS-3100, JASCO Co., Tokyo, Japan). We fixed the measuring site using the attached microscope (×100) and irradiated with a laser light and measured the Raman reflection at approximately 1500 cm^−1^ (800–1800 cm^−1^) for 10 s. The procedure was repeated 10 times, and these results were combined. We measured ten surface areas (center, near to edges, between center and edges) of each sample at random. We confirmed that all Raman peaks from the same sample were the same in wavenumbers and that the trend of the relative intensity was very similar.

## 3. Results and Discussions

### 3.1. Biofilm Formation on the Surface of Stainless Steel Using Seawater

**Figure 4:**
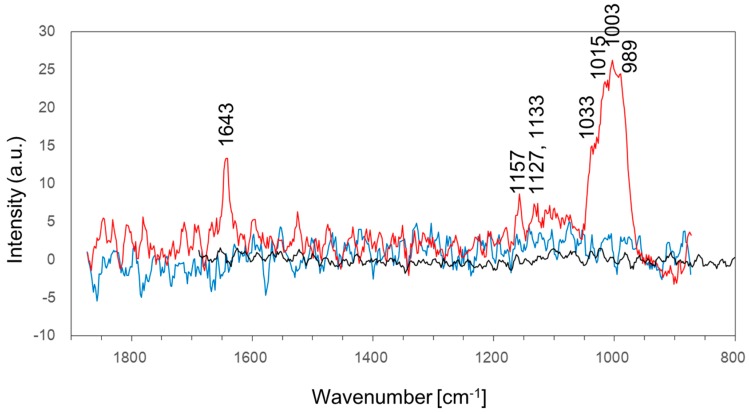
Raman spectra of stainless steel before soaking and after soaking in IB seawater or filtrated seawater. SUS304 coupons were soaked in LBR containing IB seawater (**red** line) or filtrated seawater (**blue** line) for three days. The **black** line shows the Raman spectrum of an SUS304 coupon before soaking.

We examined whether or not IB seawater would have the capacity to create a biofilm on the surface of the coupons using the LBR system. SUS304 stainless steel was placed in the system and IB seawater was circulated for three days at room temperature. IB seawater, filtered with a 0.1 μm pore size filter, was separately used as cooling water to verify that the microbes in the seawater would take part in biofilm formation. In theory, a 0.1 μm pore size filter can catch all the microbes, and, therefore, there should be no microbes in the filtered seawater. After circulation for three days, each coupon was taken out of the LBR system. The condition of the surface was examined using a high-speed microscope, and the components of the debris formed on the coupon were analyzed by Raman spectroscopy. Unfortunately, we could not obtain any morphological images of the surface of the coupons because the thickness of the debris on the coupon was so thin that we could not detect them. We have presumed that there were two reasons for that. The first one was that the exposure time might be not enough to develop the biofilm on stainless steels. In addition, the second one was that the chromium or nickel components in the oxide film formed on the stainless steel specimens might inhibit inevitably the biofilm formation in the short period. Even though biofilms were formed on the surface of silicon tubing of the LBR, all matters were derived from seawater. This is why we analyzed them by Raman spectroscopy only. Coupons soaked in IB seawater showed a broad large peak at 989–1033 cm^−1^, small peaks at 1127 cm^−1^, 1133 cm^−1^ and 1157 cm^−1^, and a sharp peak at 1643 cm^−1^ (Figure 4). These peaks are interpreted in Table 1. Conversely, coupons that were not soaked in seawater or were soaked in filtered seawater did not show any remarkable peaks. All of the detected peaks were related to the components of biofilm or microorganisms, such as polysaccharides, proteins, nucleic acids and lipids. This result indicates that the original IB seawater has the capacity to accumulate organic compounds and/or grow microbes on the surface of stainless steel, i.e., create biofilm, and that a 0.1 μm pore size filter is an effective method for removing some components that are needed to create biofilm on the surface of stainless steel.

We succeeded in developing an efficient biofilm formation system using a closed LBR system mimicking a cooling pipe system; however, the detected Raman peaks were very weak and we failed to obtain three-dimensional images of the biofilm because it was very thin. We have presumed that there were two reasons for that. The first one was that the exposure time might be not enough to develop the biofilm on stainless steels. In addition, the second one was that the chromium or nickel components in the oxide film formed on the stainless steel specimens might inhibit inevitably the biofilm formation in the short period. The purpose of this study was to evaluate the effect of metal nanoparticles-dispersed silane coating on biofilm formation. To enhance the formation of biofilm on the surface and the detection level of the Raman shift peaks, we changed the substrate of the silane coating from SUS304 to soda lime plate glass, which we have previously used to create a good biofilm [17,18].

### 3.2. Bacteria Community of IB Seawater

**Figure 5:**
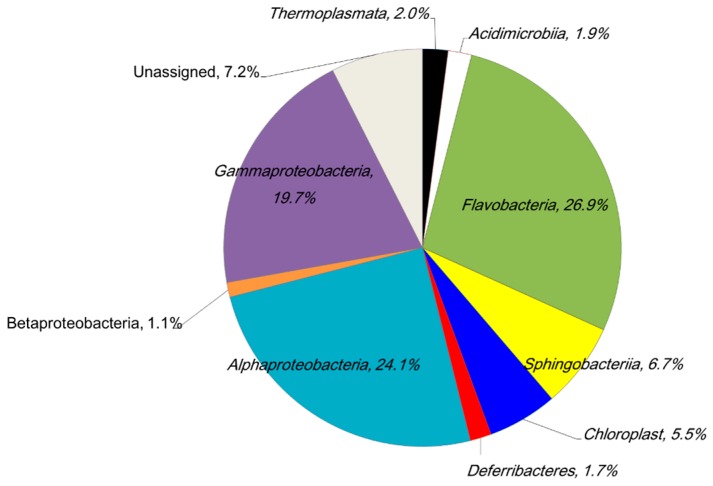
OTU content of Ise Bay seawater at a class level. The summarized data excludes classes present at less than 1%.

We have determined that IB seawater has some microbes that can take part in creating a biofilm on the surface of stainless steel. Next, we analyzed the bacterial diversity of IB seawater by *16S rRNA* gene sequencing analysis. Figure 5 shows the classification of OTU from an IB seawater sample in a class level. The most abundant group was *Flavobacteria*. In this group, *Owenweeksia*, the most abundant genus, existed at 11.9% (Table 2). *Owenweeksia* is known as the bacterium that is isolated from seawater from a 5 m of depth in Hong Kong, China, is strictly an aerobic heterotroph and requires sodium ions, magnesium ions and sea salts and either yeast extract or peptone for growth [36]. The second most abundant class was *Alphaproteobacteria*. In this group, there were three different *Rhodobacteraceae* (family, total portion was 15.3%). *Rhodobacteraceae* have been reported as the key members for initial biofilm formation in Eastern Mediterranean coastal seawater [37]. When we searched for other bacteria listed in Table 2 by the World Register of Marine spices [38], which provides the most authoritative list of names of all the marine species globally and ever published, we realized that five orders—*Acidimicrobiales*, *Oceanospirillales*, *Rickettsiales*, *Sphingobacteriales* and *Thermoplasmatales*—and six families—*Cryomorphaceae,* *Flavobacteriaceae*, *Rhodobacteraceae*, *Rhodospirillaceae*, *Alteromonadaceae* and *Oceanospirillaceae*—contained many marine-dwelling bacteria. *Tenacibaculum* (genus) was reported to live in the ocean and some of them could be cultured in seawater [39]. Summarizing marine related genera based on this information, 69.5% of OTU would be derived from marine archaea and bacteria. We collected IB seawater at a depth of 2 m and 302 m from the land in Ise Bay of Japan, which can be considered as coastal and surface seawater. Considering the result of *16S rRNA* gene analysis, we found that IB seawater has the features of surface seawater. In addition, we thought that this IB seawater had the ability to create a biofilm because it contained 15% of *Rhodobacteraceae* and that it was also good as a model of cooling water.

### 3.3. Comparison of Biofilm Formation among Several Coatings

**Figure 6:**
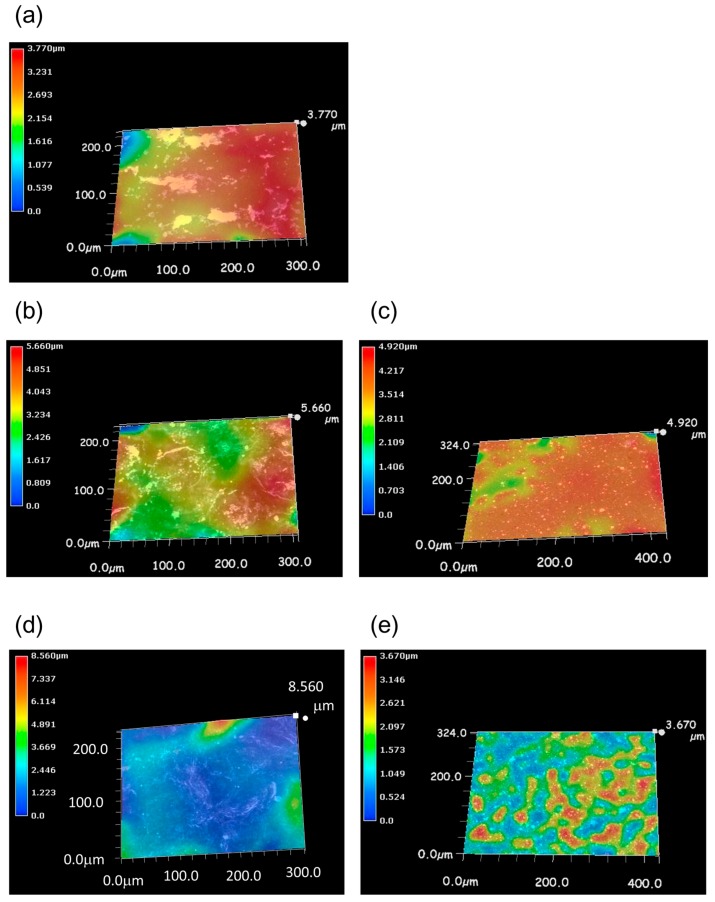
3D image of coating coupons after soaking in IB seawater. (**a**) silane coating; (**b**) 0.1 mol % Cu coating; (**c**) 5 mol % Cu coating; (**d**) 0.1 mol % Ag coating and (**e**) 5 mol % Ag coating. The **white** area shows the optical microscope image merged with the color image.

We prepared three coatings: silane coating used as a control, copper nanoparticles–dispersed silane coating (Cu coating) and silver nanoparticles–dispersed silane coating (Ag coating). In the Cu coating and Ag coating, we tried two dispersion concentrations: 0.1 mol % and 5 mol %. After soaking for three days, each coupon was taken out from the LBR and roughness of the surface was measured using a digital microscope and relative difference of the depth on the surface was visualized using color contrast. Figure 6 shows the reconstructed three-dimensional image of the surface area of each coupon. These images were merged with optical microscope images, which are shown in white. Biofilm generally forms sea-island-like structures [40] on the attached surface before reaching saturation. Therefore, if biofilm is formed on the surface, the surface image should consist of multiple colors: cold colors indicate lower level and warm colors indicate higher level, which indicates sea-island-like structure. The control coating, 0.1 mol % Cu coating and 5 mol % Ag coating showed all the colors in the images and the morphological image of 5 mol % Ag coating looked exactly like a sea-island-like structure (Figure 6a–e). Conversely, 0.1 mol % Ag coating showed most parts of area blue and other one green and slightly red (Figure 6e). 5 mol % Cu coating showed most parts of area orange and other one light green, yellow and slightly red (Figure 6c). As for results, we assumed biofilm was formed on the surface of control, 0.1 mol % Cu coating and 5 mol % Ag coating, but 5 mol % Cu coating and 0.1 mol % Ag coating were formed very little.

**Figure 7:**
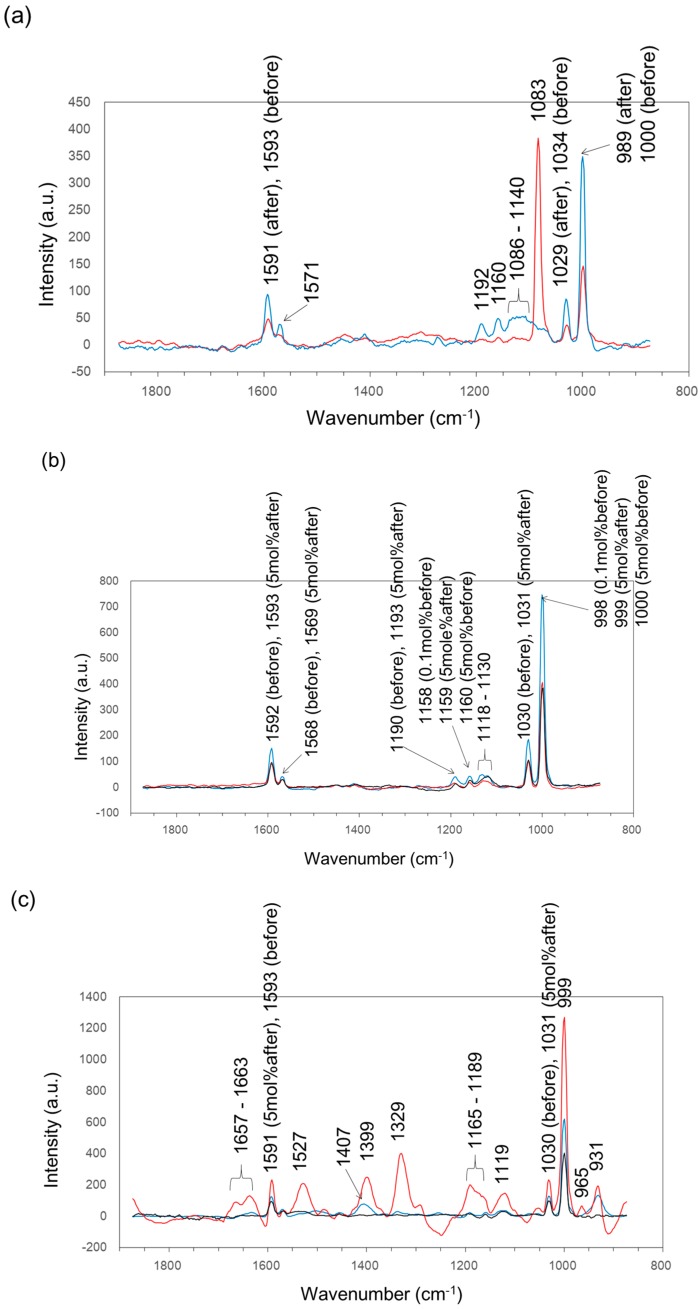
Raman spectra of three coating conditions before and after soaking in IB seawater. (**a**) silane coating: the line color indicates the mean Raman spectrum of the silane coating before (**blue** line) and after (**red** line) soaking; (**b**) Cu coating: the line color indicates the mean Raman spectrum of the 0.1 mol % Cu coating before soaking, 5 mol % Cu coating before (**blue** line) and after (**red** line) soaking; (**c**) Ag coating: the line color indicates the mean Raman spectrum of the 0.1 mol % Ag coating before soaking, 5 mol % Ag coating before (**blue** line) and after (**red** line) soaking.

We also analyzed the deposits on the surface of each coupon by Raman spectroscopy to confirm whether a biofilm was formed. Fortunately, we could detect Raman peaks from the coupons before soaking, silane coating after soaking, 5 mol % Ag coating after soaking and 5 mol % Cu coating after soaking (Figure 7). Raman spectroscopy can detect molecular vibrations, i.e., several chemical bonds of organic compounds and polymers derived from biofilm. Regardless of whether the coupons were soaked or not, several common peaks were detected in all coupons at 998–1000 cm^−1^ (strong peak), 1029–1034 cm^−1^ (medium-sized peak), 1568–1571 cm^−1^ (weak peak) and 1591–1593 cm^−1^ (medium-sized peak). The 998–1000 cm^−1^ peak and 1029–1034 cm^−1^ peak were assigned to the Si–O bond of the silane-based resin [41,42,43]. The 1568–1571 cm^−1^ peak and 1591–1593 cm^−1^ peak were assigned to the aromatic C–C stretching of the silane-based resin [44]. In addition, there were three small peaks at 1086–1140 cm^−1^, 1160 cm^−1^ and 1192 cm^−1^ in the silane coating before soaking, three small peaks at 1118–1130 cm^−1^, 1158–1160 cm^−1^ and 1190–1193 cm^−1^ in the Cu coating both before and after soaking and one medium-sized peak at 931 cm^−1^ in the Ag coating both before and after soaking. These peaks might also be related to Si–O stretching of the silane compounds that have been affected by the dispersed silver or copper [45]. These results indicate that Raman spectroscopy can be used to detect silane polymers. A very strong sharp peak was detected at 1083 cm^−1^ for the silane coating after soaking, which was presumed to derive from nucleic acids and/or mainly from the C–N stretching mode of the protein and to a minor extent from the C–N stretching mode of the lipid [33]. Several broad medium sized peaks at 1119 cm^−1^, 1165–1189 cm^−1^, 1329 cm^−1^, 1399 cm^−1^, 1527 cm^−1^, and 1637–1663 cm^−1^ were detected for the 5 mol % Ag coating after soaking. Each peak was assigned based on reference [33] as follows: the 1119 cm^−1^ peak was C–C stretching of lipid, 1165–1189 cm^−1^ was a mix of peaks derived from lipid, tyrosine, cytosine, guanine and adenine. The 1329 cm^−1^ peak was CH_3_CH_2_ wagging mode in the purine bases of nucleic acids, the 1399 cm^−1^ peak contained a C=O symmetric stretch, CH_2_ deformation and N–H in-plane deformation, derived from proteins, lipids and carbohydrates. The 1527 cm^−1^ peak was carotenoid, and 1637–1663 cm^−1^ was a mixture of amide I of the protein and the C=C bond of the lipid. We thought that proteins, carbohydrates (polysaccharides) and nucleic acids were the main components of the extracellular polymeric substance. In addition, lipids and carbohydrates (polysaccharides) would be derived from the bacterial cellular membranes and/or cellular walls. Therefore, we successfully confirmed the formation of biofilm on the surface of the control and 5 mol % Ag coating. Conversely, no peaks were detected for the 5 mol % Cu coating after soaking that were different to those for the Cu coating before soaking, which showed that biofilm was not formed on the surface of the 5 mol % Cu coating.

Some researchers have reported that silver and its nanoparticles are effective at delaying or decreasing biofilm formation [46,47,48]. In this study, a 0.1 mol % Ag coating inhibited the formation of biofilm; however, the 5 mol % Ag coating did not. After we synthesized the 5 mol % Ag-coating, we observed visually the some surface area colored light grey that might be derived from the silver. Generally, metal nanoparticles trend to cohere each other by molecular interaction. We assumed that 5 mol % silver was enough concentration to aggregate each other easily. We have to improve the technique of dispersing metal nanoparticles by changing the solvent of polymerization and/or changing the process of dispersing nanoparticles. We believe that the 5 mol % concentration of silver nanoparticles was difficult to disperse into the silane polymer so that most parts of the surface would consist of a silane coating only, i.e., there would be no silver nanoparticles. This is why only low concentrations of silver nanoparticles coating was effective at regulating the formation of biofilm. The 0.1 mol % Cu coating did not influence the formation of biofilm but the 5 mol % Cu coating strongly inhibited the formation of biofilm. Ruparelia et al. reported that copper nanoparticles inhibited the growth of *E. coli*, *Bacillus subtilis* and *Staphylococcus aureus* even though the minimum inhibitory concentration (MIC) was different (0.32 mM–4.4 mM) between the bacterial species [46]. Generally, metallic nanoparticles have a higher chemical reaction activity in comparison with bulk [49]. The 0.1 mol % Cu coating was estimated to have a concentration of approximately 10 mM, which was higher than the MIC of copper. In theory, the 0.1 mol % Cu coating should have enough concentration to inhibit the growth of several bacteria. However, in this study, the 0.1 mol % Cu coating did not affect formation of biofilm. However, the 5-mol % (about 500 mM) Cu coating inhibited the formation of biofilm. We assume that the copper nanoparticles in the Cu coating would be in a more stable state than normal copper nanoparticles because they were captured in the net structure of the silane polymers, which work as a protector that prevent the copper nanoparticles from reactions with solvent including seawater and microorganisms. As results, the 0.1 mol % Cu coating would have a lower copper concentration than the MIC, but the 5 mol % Cu coating would have a high enough copper concentration to inhibit the cell growth of a biofilm constituted of bacteria, resulting in the reducing biofilm formation.

Metal nanoparticles have more surface area and are more active than in bulk. This feature of nanoparticles results in a short effective period in which they can inhibit bacterial growth and biofilm formation. For a cooling water system, we have to reduce the bacterial growth and formation of biofilm as long as possible to reduce the costs. Dispersing nanoparticles into silane coatings is an effective method to extend the effective period to prevent the formation of a biofilm. In this case, we used a glass substrate to evaluate the coating materials free from the effect of substrates. According to the results, which we obtained in the past, we presume the positive tendency of the coating agent on glasses would be true also in the case of metallic substrate [16,17,18].

### 3.4. The Effect of Silver or Copper Dispersed Silane Coating Stainless Steel on Anti-Biofilm Formation

**Figure 8:**
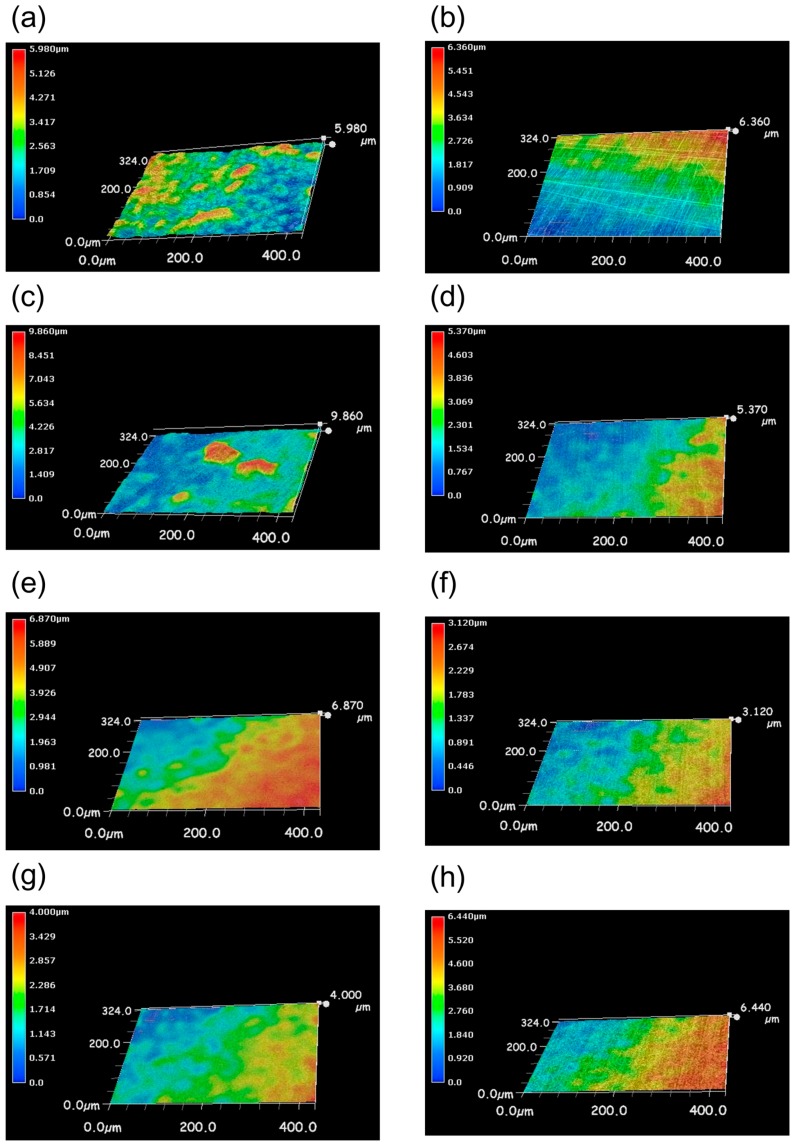
3D image of coating stainless coupons after (**left** panels) and before (**righ**t panels) soaking in IB seawater. (**a**,**b**) SUS 304; (**c**,**d**) silane coating; (**e**,**f**) 0.1 mol % Cu coating; (**g**,**h**) 0.1 mol % Ag coating. The **white** area shows the optical microscope image merged with the color image.

The silane coating was applied to stainless steels (SUS304) to determine if silver or copper nanoparticles-dispersed silane coating would be effective or not. Silane resin coating with dispersed 0.1 mol % Ag and 1.1mol % Cu was coated to stainless steels (SUS304). These coupons were incubated in the closed LBR with the seawater for seven days. After the culture, each coupon was observed with digital microscope and analyzed with Raman spectroscopy. Compared to the three-dimensional images of specimens after soaking with those before soaking, SUS304 and silane coating specimens were clearly confirmed the sea-island-like structures after soaking (Figure 8a,c, respectively). On the other hand, 0.1 mol % Cu coating and 0.1 mol % Ag coating specimens showed their inherent hubbly pattern (Figure 8f,h, respectively). Therefore, the surfaces of 0.1 mol % Cu coating and 0.1 mol % Ag coating were too difficult to be fixed as biofilm. Sea-island-like structure is the specific shape of biofilm and we are able to confirm biofilm formation by three-dimensional images. However, if a coupon has an originally hubbly surface, such as Cu coating and Ag coating, we have to analyze the surface of coupon by other methods.

**Figure 9:**
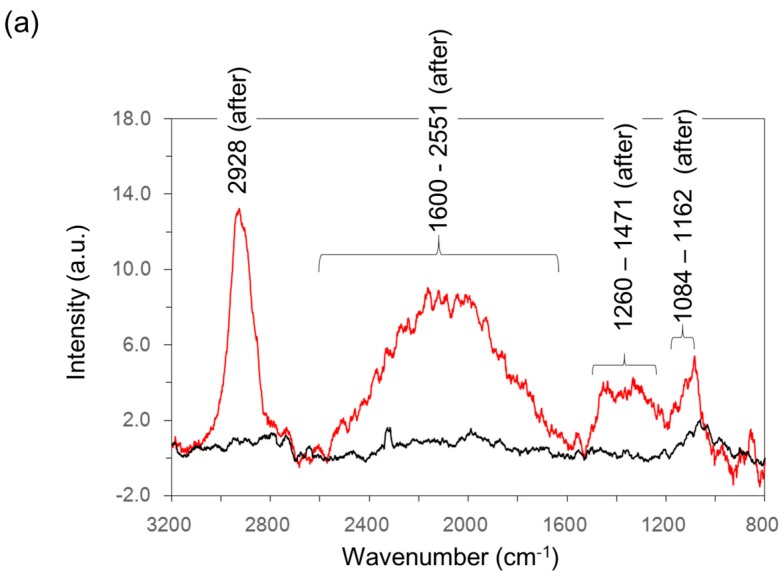

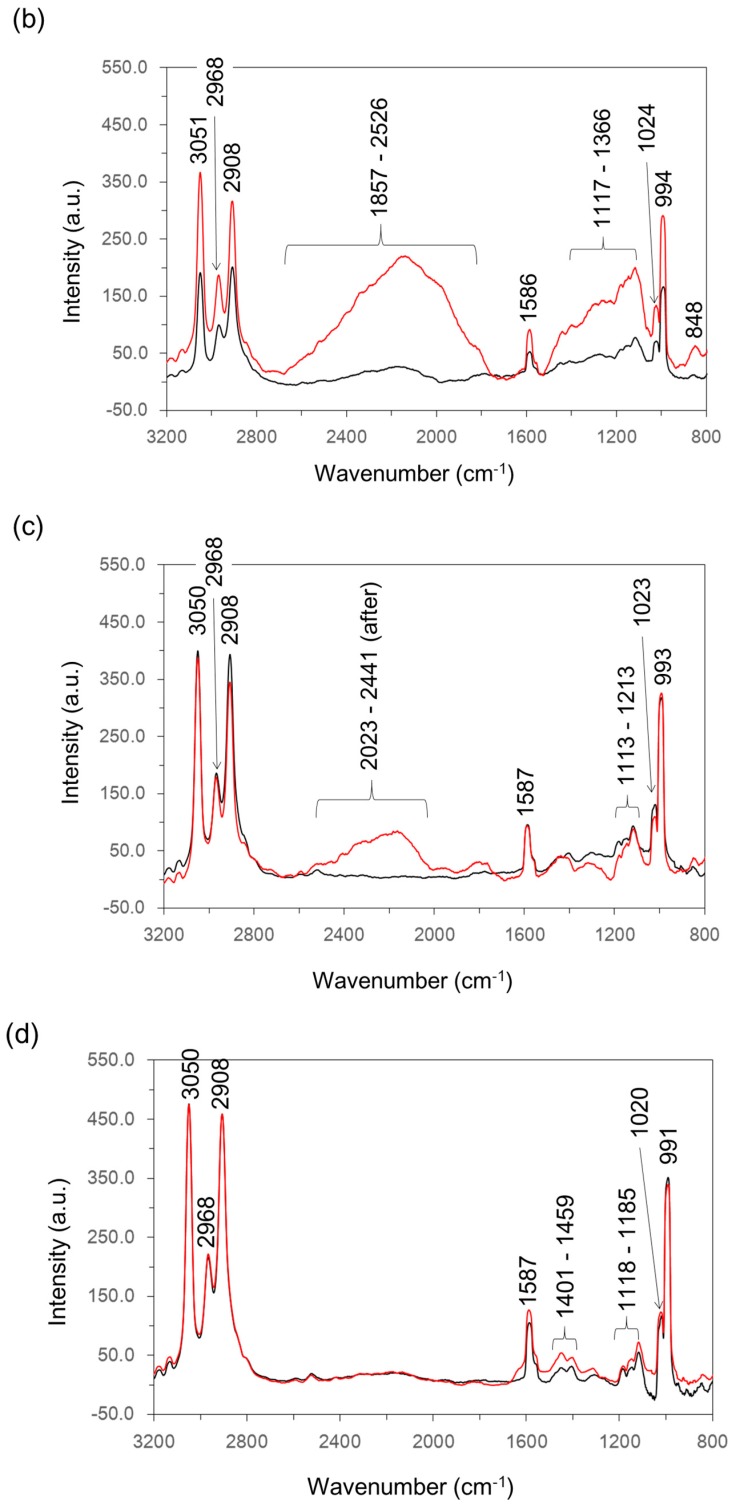
Raman spectra of coating stainless coupons before and after soaking in IB seawater. (**a**) SUS304; (**b**) silane coating; (**c**) 0.1 mol % Cu coating; (**d**) 0.1 mol % Ag coating. **Black** lines and **red** ones indicate the mean Raman spectrum of before soaking and after soaking, respectively.

Raman spectra for each coupon before and after soaking were summarized in Figure 9. As for the specimens (SUS304 and silane coating) after soaking, a strong sharp peak was detected at 2928 cm^−1^ derived from fatty acid stretching vibration of C=H_3_ [50]. In addition, four broad peaks at 1084–1162 cm^−1^, 1260–1471 cm^−1^ and 1600–2551 cm^−1^ were assigned to fatty acid stretching vibration of C–C bond, HC=CH bond of unsaturated fatty acids, mixture of (1) vibration of C=C or C=O bond of fatty acids [50]; (2) amide bond of protein [16] and (3) DNA strand bonds [8], respectively. As for silane coating, some peaks were detected at the same wave numbers with those for both before and after soaking, which were at 848 cm^−1^, 994 cm^−1^, 1024 cm^−1^, 1117–1366 cm^−1^, 1586 cm^−1^, 2908 cm^−1^, 2968 cm^−1^ and 3051 cm^−1^. The peaks at 848 cm^−1^, 994 cm^−1^, 1024 cm^−1^ were assigned to the Si–O bond of the silane-based resin [41,42,43]. The 1586 cm^−1^ peak was assigned to the aromatic C–C stretching of the silane-based resin [44]. Unfortunately, there were no references for the peaks over 2900 cm^−1^. However, we presume that they could be derived from silane-based resin, since the portion of relative intensity was similar between the specimens before and after soaking. On the other hand, the two large broad peaks were detected at 1117–1366 cm^−1^ and 1857–2526 cm^−1^ for the specimens after soaking. The former was assigned to the mixture of C–N vibration of protein and C–C bond of lipid, and the latter was assigned to the mixture of vibration of C=C or C=O bond of fatty acids [50], amide bond of protein [16] and DNA strand bonds [8]. About 0.1 mol % Cu coating, peaks at 993 cm^−1^, 1023 cm^−1^, 1113–1213 cm^−1^, 1587 cm^−1^, 2908 cm^−1^, 2968 cm^−1^ and 3050 cm^−1^ were derived from silane-based resin as silane coating. Only the peak at 2023–2441 cm^−1^ was detected for the specimen after soaking, which was assigned to be the mixture of amide bond of protein [16] and DNA strand bonds [8]. At about 0.1 mol % Ag coating, all detected peaks were related to silane-based resin (peaks at 991 cm^−1^, 1020 cm^−1^, 1118–1185 cm^−1^, 1587 cm^−1^, 2908 cm^−1^, 2968 cm^−1^ and 3050 cm^−1^). As mentioned in previous sections, proteins, nucleic acids (DNA) and lipids are the main components of biofilm. The Raman peaks related to proteins, nucleic acids and lipids were detected for the specimen (SUS304) just after soaking, silane coating and 0.1 mol % Cu coating. Compared with the relative intensity of the peaks at 2968 cm^−1^ (derived from silane-based resin), silane coating (peak at 1857–2526 cm^−1^) was almost the same, while the 0.1 mol % Cu coating (2023–2441 cm^−1^) was half. From these results, we could presume that biofilms were formed on the surface of SUS304, silane coated stainless steel and the specimen coated with 0.1 mol % Cu dispersed silane. As for 0.1 mol % Ag dispersed stainless steel, no traces for biofilm could not be found. However 0.1 mol % Cu coating could control biofilm formation to some extent. In conclusion, both 0.1 mol % Ag coating and 0.1 mol % Cu coating will be able to inhibit or delay the biofilm formation on cooling pipe systems, and 0.1 mol % Ag coating will be more effective for anti-biofilm formation than that of 0.1 mol % Cu coating.

## 4. Conclusions

In this study, we proposed metal nanoparticle-dispersed silane coatings to inhibit the formation of biofilm in cooling pipe systems. Copper nanoparticle-dispersed silane coatings succeeded in inhibiting biofilm formation when the copper concentration was 5 mol %. In addition, silver nanoparticles-dispersed silane coatings also affected the inhibiting biofilm formation when the silver concentration was 0.1 mol %. However, 5 mol % of silver dispersed silane coating was not effective at preventing the formation of biofilm because silver nanoparticles aggregated each other, and we could not make uniform silver nanoparticle-dispersed silane coating. We found that the dispersion of silver or copper nanoparticles in silane coatings is a powerful method for reducing biofilm formation in cooling pipes to produce metallic ions more easily and also to increase environmental safety without any organo-metals. However, more reliable dispersion techniques are needed to take advantage of these properties.

## Figures and Tables

**Table 1:** The spectral interpretation of SUS304 soaked in IB seawater.

Wavenumber (cm^−1^)	Assignment	Reference
989	phosphate ion stretching vibration	[33]
1003	phenylalanine	[34]
1015	carbohydrates peak for solids	[34]
1033	C–H in-plane phenylalanine of proteins n(CO), n(CC) and n(CCO) of polysaccharides or pectin	[34]
1127	ν(C–N)	[34]
1133	palmitic acid and fatty acid	[34]
1153	C–C bond of lipid	[34]
1643	amide I bond of the protein	[35]

**Table 2:** OTU content of Ise Bay seawater at a general level. The ratio of OTU was summarized only above 1%. Unassigned OTU occupied 7.2%.

Domain	Phylum	Class	Order	Family	Genus	Abundance (%)
*Archaea*	*Euryarchaeota*	*Thermoplasmata*	*Thermoplasmatales*	*Marine_Group_II*	-	2.0
*Bacteria*	*Actinobacteria*	*Acidimicrobiia*	*Acidimicrobiales*	*OCS155_marine_group*	-	1.8
	*Bacteroidetes*	*Flavobacteria*	*Flavobacteriales*	*Cryomorphaceae*	*Owenweeksia*	11.9
	*Bacteroidetes*	*Flavobacteria*	*Flavobacteriales*	*Flavobacteriaceae*	*Tenacibaculum*	4.7
	*Bacteroidetes*	*Flavobacteria*	*Flavobacteriales*	*Flavobacteriaceae*	*NS5 marine group*	4.4
	*Bacteroidetes*	*Flavobacteria*	*Flavobacteriales*	*Flavobacteriaceae*	*NS4 marine group*	4.2
	*Bacteroidetes*	*Sphingobacteriia*	*Sphingobacteriales*	*NS11-12_marine_group*	-	6.3
	*Cyanobacteria*	*Cyanobacteria*	*Chloroplast*	*Chloroplast*	*Chloroplast*	5.5
	*Proteobacteria*	*Alphaproteobacteria*	*Rhodobacterales*	*Rhodobacteraceae*	*Roseobacter clade OCT lineage*	5.4
	*Proteobacteria*	*Alphaproteobacteria*	*Rhodobacterales*	*Rhodobacteraceae*	-	5.3
	*Proteobacteria*	*Alphaproteobacteria*	*Rhodobacterales*	*Rhodobacteraceae*	*Roseobacter_clade NAC11-7 lineage*	4.8
	*Proteobacteria*	*Alphaproteobacteria*	*Rhodospirillales*	*Rhodospirillaceae*	*AEGEAN-169 marine group*	1.2
	*Proteobacteria*	*Alphaproteobacteria*	*Rickettsiales*	*SAR116 clade*	-	1.7
	*Proteobacteria*	*Alphaproteobacteria*	*SAR11 clade*	*Surface_1*	-	3.6
	*Proteobacteria*	*Gammaproteobacteria*	*Alteromonadales*	*Alteromonadaceae*	*OM60(NOR5) clade*	4.7
	*Proteobacteria*	*Gammaproteobacteria*	*Alteromonadales*	*Alteromonadaceae*	*SAR92_clade*	1.0
	*Proteobacteria*	*Gammaproteobacteria*	*KI89A clade*	-	-	1.6
	*Proteobacteria*	*Gammaproteobacteria*	*Oceanospirillales*	*Oceanospirillaceae*	*Pseudospirillum*	1.5
	*Proteobacteria*	*Gammaproteobacteria*	*Oceanospirillales*	*SAR86 clade*	-	1.1
	*Proteobacteria*	*Gammaproteobacteria*	*Oceanospirillales*	*ZD0405*	-	7.5
